# Targeting V-ATPase Isoform Restores Cisplatin Activity in Resistant Ovarian Cancer: Inhibition of Autophagy, Endosome Function, and ERK/MEK Pathway

**DOI:** 10.1155/2019/2343876

**Published:** 2019-04-01

**Authors:** Arpita Kulshrestha, Gajendra K. Katara, Safaa A. Ibrahim, Valerie Riehl, Manoranjan Sahoo, James Dolan, Kyle W. Meinke, Michael R. Pins, Kenneth D. Beaman

**Affiliations:** ^1^Department of Microbiology and Immunology, Rosalind Franklin University of Medicine and Science, North Chicago, IL, USA; ^2^Department of Microbiology and Immunology, Faculty of Pharmacy, Cairo University, Egypt; ^3^Department of Obstetrics & Gynecology, Advocate Lutheran General Hospital, Park Ridge, IL, USA; ^4^Department of Pathology, Advocate Lutheran General Hospital, Park Ridge, IL, USA

## Abstract

Ovarian cancer (OVCA) patients often develop tolerance to standard platinum therapy that accounts for extensive treatment failures. Cisplatin resistant OVCA cells (cis-R) display enhanced survival mechanisms to cope with therapeutic stress. In these cells, increased autophagy process assists in chemoresistance by boosting the nutrient pool under stress. To improve the treatment response, both protective autophagy inhibition and its overactivation are showing efficacy in chemosensitization. Autophagy requires a tightly regulated intracellular pH. Vacuolar ATPases (V-ATPases) are proton extruding nanomotors present on cellular/vesicular membranes where they act as primary pH regulators. V-ATPase ‘a2' isoform (V0a2), the major pH sensing unit, is markedly overexpressed on the plasma membrane and the early endosomes of OVCA cells. Previously, V0a2 inhibition sensitized cis-R cells to platinum drugs by acidifying cytosolic pH that elevated DNA damage. Here, we examined how V0a2 inhibition affected endosomal function and the autophagy process as a possible factor for cisplatin sensitization. Clinically, V0a2 expression was significantly higher in tissues from drug nonresponder OVCA patients compared to treatment responders. In vitro V0a2 knockdown in cis-R cells (sh-V0a2-cisR) significantly reduced the tumor sphere-forming ability and caused complete disintegration of the spheres upon cisplatin treatment. The apoptotic capacity of sh-V0a2-cisR improved substantially with potentiation of both intrinsic and extrinsic apoptotic pathway when treated with cisplatin. Unlike the chemical V-ATPase inhibitors that acutely induce autophagy, here, the stable V0a2 inhibition dampened the protective autophagy process in sh-V0a2-cisR cells with downregulated expression of proteins beclin-1, ATG-7, and LC3B and low autophagosome numbers compared to control cis-R cells. These cells showed downregulated ERK/MEK pathway that is known to repress autophagy. Interestingly, upon cisplatin treatment of sh-V0a2-cisR, the autophagy initiation proteins (LC3B, ATG7, and Beclin 1) were found upregulated as a stress response compared to the untreated cells. However, there was a concomitant downstream autophagosome accumulation and an enhanced P62 protein levels indicating the overall block in autophagy flux. Mechanistically, V0a2 knockdown caused defects in early endosome function as the transferrin internalization was impaired. Taken together, this study provides a novel insight into the mechanism by which V-ATPase-isoform regulates autophagy that assists in chemoresistance in ovarian cancer. We conclude that V-ATPase-V0a2 is a potent target for developing an effective treatment to enhance patient survival rates in ovarian cancer.

## 1. Introduction

Ovarian cancer (OVCA) is hard to treat as it exhibits refractoriness to standard chemotherapy approaches including platinum-based drugs [1]. In addition to apoptosis inhibition, cisplatin resistant cancer cells rely on mechanisms such as reduced drug uptake, increased drug efflux, enhanced DNA-repair, and defective signaling pathways to survive therapeutic cell death [2]. Nevertheless, an understanding of the precise molecular mechanism of chemoresistance will help design strategies to improve the treatment outcome in OVCA patients.

Exposure of cancer cells to cisplatin elicits a stress response which induces coping mechanisms that favor cancer cell survival [3]. Autophagy is the primary protective process that enables energy supply during stress such as chemotherapy exposure and nutrient depletion [4–6]. The self-degradative pathway of autophagy involves the formation of double-membrane vesicles (autophagosomes) around damaged cellular proteins and organelles [7, 8]. Autophagosomes fuse to endo-lysosomal machinery where sequestered cellular components are ultimately digested for energy recycling [9]. In addition to lysosomal machinery, recent studies suggest the importance of early endosomes in autophagy [10]. It is therefore important to understand how molecular targets involved in endosomal machinery can modulate autophagy process.

A tightly regulated intracellular pH is critical for autophagy [11]. In mammalian cells, vacuolar ATPase (V-ATPase) proton pumps are the primary pH regulators that maintain intravesicular and/or extracellular pH. In normal cells, V-ATPases pump protons from the cytoplasm to the lumen of the acidic organelles [9]. In cancer cells, plasma membrane-associated V-ATPases extrude protons and acidify the extracellular matrix [12, 13]. V-ATPase inhibition disrupts tumor pH gradients that alters drug retention and trafficking in tumor cells. Many proton pump/V-ATPase inhibitors are showing efficacy in increasing the sensitivity of tumor cells to cytotoxic agents [14–16]. Unlike chemical inhibitors, targeting cancer specific V-ATPase isoforms will modulate autophagy and will potentially decrease the associated toxicity to normal cells. Our previous work highlighted that, in OVCA cells, ‘a2' isoform (V-ATPase-V0a2) is overexpressed in cisplatin resistant cells and is a component of plasma-membrane V-ATPase and the early endosomal machinery [17, 18]. Inhibition of V-ATPase-V0a2 acidified the cytosol thereby sensitizing the resistant OVCA cells to platinum mediated DNA damage [18]. However, it is not known how V-ATPase-V0a2 regulates cisplatin sensitivity through the endosome dependent autophagy process.

Here, we investigated the relationship between V-ATPase inhibition, cisplatin sensitization, and the autophagy process. We provide evidence that, in chemoresistant OVCA cells (cis-R), inhibition of V-ATPase-V0a2 blocks the autophagy flux and suppresses ERK/MEK pathway that promotes cisplatin-mediated cell death. Our findings provide a rationale for the utility of V-ATPase-V0a2 inhibitors in combination with standard drugs as a novel strategy to improve the treatment efficacy of the chemoresistant ovarian cancer.

## 2. Material and Methods

### 2.1. Cell Lines and Cell Culture

Human ovarian carcinoma cell line A2780 (Sigma–Aldrich), its acquired cisplatin resistant counterpart cis-A2780, and TOV-112D cell lines were employed in this study as described previously [17, 18]. Briefly, A2780 and cis-A2780 cells were cultured in RPMI 1640 medium (Invitrogen, Carlsbad, CA) supplemented with 10% (v/v) heat-inactivated fetal bovine serum (Biowest LLC, MO, USA), 100 U/ml penicillin, and 100 U/ml streptomycin (Sigma–Aldrich) at 37°C, 5% CO2. TOV112D cell line (American Type Culture Collection [ATCC], Manassas, VA) was cultured in CTOV medium [1:1 mixture of MCDB 105 medium containing a final concentration of 1.5 g/L sodium bicarbonate and medium 199 containing a final concentration of 2.2 g/L sodium bicarbonate at 37°C, 5% CO2]. The cells were routinely grown until reaching 80% confluency and then subcultured or plated for experiments.

### 2.2. Generation of Stable V-ATPase-V0a2 Knockdown Cells

The shRNA mediated V-ATPase-V0a2 knockdown was performed as described previously [18]. Briefly, the cisplatin resistant cells (cis-A2780) were plated overnight and then transfected with V0a2 shRNA constructs (Suresilencing Plasmid, Qiagen, Valencia, CA, USA) or a scrambled control shRNA using the Attractene transfection reagent (Qiagen). The cells were treated with the selection antibiotic (1 mg/ml G418) after 24 h after transfection. Medium containing G418 was replenished every 72 h. After confirming the knockdown by Q-RT PCR, the positive transfectants were cloned and used for further experiments.

### 2.3. Drugs

Anticancer drug cisplatin was obtained from Sigma–Aldrich. Cisplatin 1mM stock was prepared in normal saline (0.9% NaCl) and stored as aliquots at -20°C up to 3 months. V-ATPase inhibitor bafilomycin A (Sigma–Aldrich, M17931) was dissolved in DMSO at a 100*μ*M stock solution. Autophagy modulators rapamycin and chloroquine were procured from Enzo Life Sciences, USA. Rapamycin was dissolved in DMSO at a 500*μ*M stock solution. Chloroquine was dissolved in deionized water for a 60 mM stock solution. For long term storage, all stock solutions were stored at -20°C. Selective MEK inhibitor cobimetinib (10mM in DMSO) was obtained from ApexBio and the stock solutions were stored at -20°C.

### 2.4. RNA Isolation and Reverse Transcription-PCR

The OVCA cells were washed with PBS and detached using accutase solution (Sigma–Aldrich, St Louis, MO, USA). For RNA extraction, RNeasy® mini kit (Qiagen, Valencia, CA) was used according to the manufacturer's instruction. Reverse transcription was performed using the high capacity cDNA kit (Applied Biosystems, Foster City, CA) according to manufacturer's protocol. All real-time PCR reactions were performed in triplicate in 10 *μ*l volume using Universal fast PCR Master Mix reagent (Applied Biosystems, USA) according to the manufacturer's instructions. The results were analyzed using the ΔΔCt method using GAPDH as the endogenous control. For cell death and autophagy pathway analysis (RT2 profiler, SA Biosciences, Frederick, MD, USA), PCR array-based expression profiling was performed using SYBR-Green method and the results were analyzed using the ΔΔCt method using RT^2^ profiler PCR data analysis software version 3.5 (SA Biosciences).

### 2.5. Antibodies

The following primary antibodies were employed in study: rabbit anti-GAPDH [1:400; Cell Signaling Technology (CST); Catalog number-5174S)], rabbit anti-LC3B (1:400; CST; 2775S), rabbit anti-beclin1 (1:350; CST; 5174S), rabbit anti-ATG7 (1:400; CST; 8558S), rabbit anti-P62 (1:400; CST; 5114S), anti-cleaved caspase 8 (1:200; CST; 9748), anti-phospho BRAF (1:1000; CST; 2696T), anti-phospho-MEK (1:400; CST; 9154T), Mouse anti-beta actin (1:10,000; Abcam; ab184220), rabbit anti-LAMP-1(1:250; Abcam; ab25630), rat anti-LAMP-2 (1:250; Abcam; ab25631), rabbit anti-Fas L (1:100; Abcam; ab15285), rabbit anti-caspase 3 (1:400; Thermo Fisher; 4331182), rabbit anti-Fas (1:100; Bio legend; 305611), and mouse anti-a2V (Covance, Denver, USA). For isotype-control antibodies, control mouse IgG (R&D Systems) and rabbit IgG isotype (Invitrogen) were used. Secondary antibodies were as follows: goat anti-rabbit IgG-FITC, donkey anti-mouse IgG AF-594, donkey anti-rabbit IgG AF-594 (Invitrogen), rabbit anti-rat IgG-FITC (Abcam), donkey anti-rabbit IRDye-800CW, and donkey anti-mouse IRDye-680 CW (LI-COR Bioscience, Lincoln, NE).

### 2.6. Immunohistochemical Staining of Ovarian Cancer Tissue

To explore the clinical relevance of V0a2 expression in modulating cisplatin efficacy, we obtained paraffin-embedded tissues from ovarian cancer patients who reported to Advocate Lutheran General Hospital (ALGH), Chicago, USA. The study was approved by the Ethics Committee of ALGH. Eight samples each from the drug responder and nonresponder patient group were selected. 5-*μ*m serially sectioned slides were prepared. For normal control tissues, ovarian tissue sections from the normal ovary (n=2) were obtained from Biochain Institute, Inc. (Newark, CA, USA). The horseradish peroxidase-labeled polymer (EnVision+Dual Link System-HRP; DAKO, USA) based staining method was used according to the manufacturer's protocol. For antigen retrieval, the sections were boiled in sodium citrate buffer (pH = 6.0) as described previously [18]. The slides were then cooled, blocked with 5% BSA in PBS, and incubated with the primary antibody at 4°C overnight. Concurrently, for negative mouse isotype-control antibody (R&D systems, USA) was used. The anti-rabbit/mouse secondary antibody was then added for 15 min at 37°C. The sections were counterstained with Mayer's hematoxylin and mounted in Faramount aqueous mounting medium (Dako). The immunostaining was evaluated by light photomicroscopy (Leica ICC50 W, USA) using a high-resolution camera.

The IHC scoring was performed using the semiquantitative integration method. In this method, five random fields of view were selected for each specimen at high magnification (×200). The following criteria were employed to generate a score: first, staining area score [SAS] (≤1%: 0; 2–25%: 1; 26–50%: 2; 51–75%: 3 and >75%: 4); second, staining intensity [SI] (light brown: 1; moderate brown: 2 and tan: 3). The IHC score was calculated using the formula: IHC score= SAS X SI.

### 2.7. Western Blot Analysis

The harvested cell pellets were resuspended in NP-40 lysis buffer containing protease and phosphatase inhibitors (Pierce Protein Biology, USA) and incubated at 4°C for 30 min, after which the cells were centrifuged at 13,000 × rpm at 4°C. The supernatant was then collected. Protein quantification was performed using the BCA assay (Pierce Protein Biology, USA). The 30 *μ*g protein lysates were boiled with 4X SDS sample buffer containing 2-mercaptoethanol and proteins were separated by SDS-PAGE on 4–20% gradient acrylamide gels. All primary antibody incubations were performed 1h at room temperature followed by secondary antibody incubation (IR dye, Licor) for 1 h at RT. The Blots were scanned using the Odyssey® infrared imaging system (LI-COR Biotechnology Lincoln, NE, USA). Blots were probed with a *β*-actin endogenous control antibody to confirm equivalent protein loading (Abcam, USA).

### 2.8. Immunofluorescence Analysis

For immunofluorescence analysis, the cells were plated in 8-well chamber slides (Nunc, USA) at 3000 cells/well and were incubated overnight at 37°C, 5% CO2. The cells were then washed thrice with PBS (containing 0.5% FBS), fixed with 4% paraformaldehyde for 30 min at room temperature (RT), and permeabilized with 0.1% Triton X-100 in PBS for 12 min, 4°C. Blocking was performed using 3% FBS in PBS for 1 h at RT. The cells were then incubated with primary antibodies (in blocking buffer) for 1h at RT. The cells were then rinsed thrice with PBST and incubated with secondary antibodies: Alexa Fluor® 488-conjugated goat anti-rabbit or Alexa Fluor® 594-conjugated goat anti-rabbit secondary antibody (1:200 dilution) (Invitrogen) dissolved in 3%FBS in PBS for 1 h at RT. The cells were prepared for viewing using ProLong® Gold (Invitrogen) mounting medium containing DAPI and allowed to polymerize at room temperature for 24 h. For confocal microscopy, the stained cells were imaged on an Olympus Fluoview Fv10i confocal microscope. The analysis was performed using Fv10i Flouview Ver.3.0 software. Experiments were repeated at least twice in duplicate. For immunofluorescence microscopy, stained cells were imaged in Olympus microscope and analyzed using NIS-Elements software (Nikon Inc., NY, USA).

### 2.9. Flow Cytometry Analysis

Sh-V0a2 transfected/untransfected cells (2.5 x10^5^ cells/tube) were washed with HBSS containing 0.1% FBS. For surface staining, the cells were incubated with mouse monoclonal FasL or Fas antibody conjugated to A_647_ or A_488_ (Covance, Denver, PA) in PBS for 40 min at RT. For the intracellular staining, the cells were fixed and permeabilized using fixation and permeabilization buffer (BD Biosciences, San Jose, CA, USA) and the cells were stained as described above. For the indirect staining, the cells were incubated with unconjugated antibodies (caspase-8) for 1 h at RT and subsequently washed twice with PBS and then stained with conjugated secondary antibody (Abcam, USA) for 30 min at RT. Appropriate isotype and unstained controls were used for the experiments. The stained cells were analyzed on a BD LSR II flow cytometer with FlowJo software (Tree Star). Experiments were performed at least twice in duplicate.

### 2.10. Assessment of Autophagosomes

For autophagy analysis, V0a2 shRNA transfected/untransfected OVCA cells were incubated with 20*μ*M cisplatin for 24h at 37°C in 5% CO2. To determine the induction of autophagy, we used the Cyto-ID Autophagy detection kit (Enzo Life Sciences, Raamsdonksveer, The Netherlands). The autophagy detection is based on monodansylcadaverine dye that specifically stains autophagosomes. For positive autophagy controls, the cells were treated with mTOR inhibitor rapamycin (0.5 *μ*M) or lysosomal alkalizer chloroquine (60*μ*M) or V-ATPase inhibitor bafilomycin (50nM). No treatment control wells were also included in each set of experiment. After 24 h, the cells were washed with assay buffer provided by the manufacturer (supplemented with 5% FBS) and stained with the Cyto-ID green detection reagent for 30 minutes and subsequently washed twice again with assay buffer. The fluorescence signal was immediately captured in LSRII flow cytometer measuring the intensity in 10,000 cells. FlowJo software was used to process the imaging data.

### 2.11. Transferrin Internalization Assay

As a measure of early endosomal function, the cellular internalization of A_594_-labeled transferrin (Tfn) was assayed. First, the cells were serum starved by rinsing with 37°C HBSS (Invitrogen, USA) and incubated in serum-free RPMI containing 25mM HEPES and 1% BSA (RPMI-BSA) for 30 minutes at 37°C, 5% CO_2_. Cells were incubated in ice for 10 minutes and then incubated in RPMI-BSA containing 50 *μ*g/ml of Tfn-A_594_ conjugate to allow internalization for upto 30 minutes. Finally, cells were quick rinsed at least 10 times with HBSS to remove surface labeling. The slides were fixed in 4% formaldehyde for 15 minutes at room temperature. Immune-fluorescence analysis for early endosome labeling (EEA1) was performed as described above. The slides were then processed for fluorescence microscopy.

### 2.12. Cell Cytotoxicity Assay

OVCA cells were seeded into 96-well plate (10,000 cells/well) overnight. The OVCA cells were exposed to cisplatin (0.5, 1, 2.5, 5, 10, 20, and 50 *μ*M) and 10nM cobimetinib (MEK inhibitor) for 48h at 37°C in 5% CO_2_. After incubation, in vitro cell viability was measured using MTS reagent (Promega, USA). Untreated cells were used as negative control. All experiments were performed in triplicate. The semilog plots of dose-response curves were generated using Microsoft Excel (Microsoft).

## 3. Statistical Analysis

The means of two data sets were compared and significance was determined by two-tailed Students t-test or Mann–Whitney U test. Differences were considered to be statistically significant where p<0.05. The data were graphically represented as the mean ± standard deviation of the mean (SD). The data were analyzed using GraphPad Prism (version 5) statistical software. All experiments were repeated at least twice in duplicate.

## 4. Results

### 4.1. V-ATPase V0a2 Is Highly Expressed in Cisplatin Nonresponder Human Ovarian Cancer Tissues

**Figure 1:**
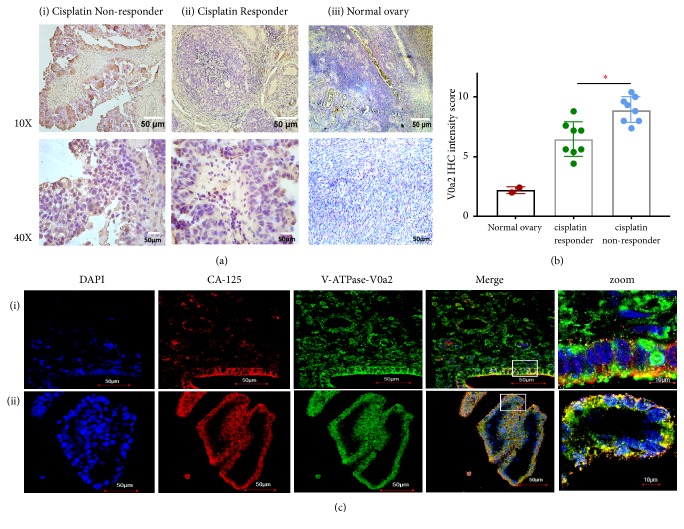
*V-ATPase-V0a2 is highly expressed in cisplatin nonresponder ovarian cancer tissues.* (a) Immunohistochemical analysis of V-ATPase-V0a2 expression in tissues from (i) cisplatin nonresponder and (ii) cisplatin responder ovarian cancer patients compared to (iii) normal human ovary tissue. Original magnification × 100 (upper panel) and X 400 (lower panel). (b) The quantitative IHC data expressed as IHC intensity score revealed higher V0a2 expression in ovarian cancer tissues from cisplatin nonresponder patients compared to responder patients and to normal ovarian tissues. (c) Confocal microscopy analysis of V0a2 (green) in nonresponder OVCA tissues ((i) and (ii)) shows its coexpression with ovarian cancer cell marker CA125 (red). Nuclear DAPI staining in blue. Merged areas are shown in yellow. Original magnification: × 600. Zoomed areas represent white boxes in merged figures. Representative images from three independent experiments are shown.

To elucidate the clinical relevance of V-ATPase-V0a2 in OVCA with varying cisplatin sensitivity, we utilized archival OVCA tissues from cisplatin responder (n=8) and nonresponder patients (n=8). The OVCA tissue samples were obtained from patients that were intrinsically responsive/nonresponsive to cisplatin (before any treatment). Normal ovarian tissues (n=2) were employed as the control. Both cisplatin responder and nonresponder patient tissues exhibited high V-ATPase-V0a2 staining compared to the normal ovary tissues. The V0a2 protein expression was higher in cisplatin nonresponder (IHC score=8.9±1.06) than cisplatin responder patient tissues (IHC score= 6.5 ± 1.4, p=0.02) or normal ovarian tissues (IHC score=2.2±0.28; p<0.01) by IHC analysis [Figures 1(a) and 1(b)]. The V-ATPase-V0a2 expression thus correlates with drug unresponsiveness in ovarian cancer patients. Our previous study showed a very poor expression of V-ATPase-V0a2 in normal ovary tissues suggesting that V0a2 expression is selectively upregulated during tumorigenesis [17]. Confocal microscopy analysis showed coexpression of V0a2 with OVCA antigen marker, CA125, confirming V0a2 expression specifically in OVCA cells in drug nonresponder tissues [Figures 1(c)(i) and 1(c)(ii)] as well as drug responder tissues [Supplementary Figure S1]. These data suggest that V0a2 is a prominent target in ovarian cancer patients with varying cisplatin sensitivity. Further studies employing tissues from relapse/posttreatment patients and including more number of samples will further improve our understanding of V-ATPase role in cisplatin resistance in OVCA.

### 4.2. Cisplatin Resistant Ovarian Cancer Cells Show Low Sphere-Forming Ability upon V-ATPase-V0a2 Inhibition

To investigate the possible association of V0a2 with cisplatin resistance in OVCA, we next performed in vitro assays employing the sh-V0a2-cisR and sh-scr-cisR control cells. Our previous study showed that stable V-ATPase-V0a2 knockdown cells exhibited 3.8-fold inhibition at mRNA level and a 2.5-fold reduction in protein expression compared to sh-scr-cisR control cells [18].

**Figure 2:**
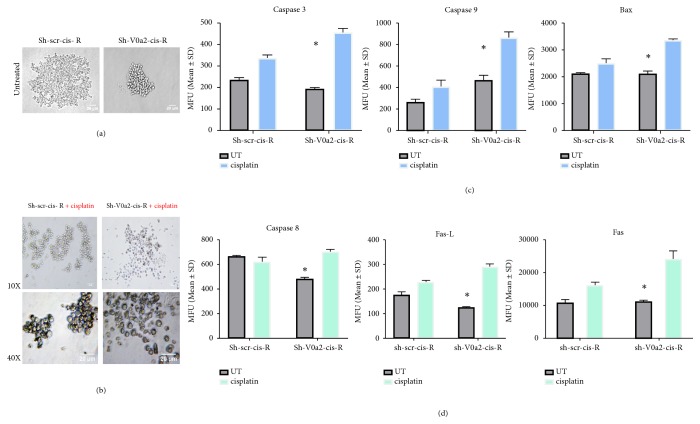
*Inhibition of V-ATPase-V0a2 in resistant ovarian cancer cells blunts spheroid formation and enhances cisplatin-mediated cell death.* (a) Photomicrographs showing the effect of shRNA mediated V-ATPase-V0a2 inhibition on the spheroid formation in cisplatin resistant ovarian cancer cells (sh-V0a2-cisR) compared to control cells (sh-scr-cis-R). The sh-V0a2-cisR exhibited decreased tumor spheroid formation while control cells formed large clusters of spheroids. (b) Upon cisplatin treatment (20*μ*M, 48h), an enhanced spheroid dissociation was observed in sh-V0a2-cisR compared to control spheroids. Original magnification: X100, X400. (c) Geometric mean fluorescence intensity of effector apoptotic protein (cleaved caspase-3), intrinsic apoptotic (active caspase-9, Bax), and (d) of extrinsic apoptotic proteins (cleaved caspase-8, Fas, and FasL) in cisplatin treated sh-V0a2-cisR compared to cisplatin treated sh-V0a2-cisR cells as quantitated by flow cytometry. Each value represents the mean ± SD of three independent experiments, *∗*P < 0.05.

3D cancer cell spheroids mimic both* in vivo* architecture and low drug penetration properties that represent a relevant model for studying drug resistance [19]. Here, we found that, upon V0a2 inhibition, sh-V0a2-cisR cells exhibited a reduced sphere-forming ability compared to control OVCA cells (sh-scr-cis-R) [Figure 2(a)]. Further, upon cisplatin treatment (20*μ*M, 48h), we observed dissociation of sh-V0a2-cisR spheroids while control spheroids remained unaffected [Figure 2(b)]. There was no difference in the sphere-forming ability between cisplatin sensitive (A2780) and cisplatin resistant cells (cis-A2780; data not shown).

### 4.3. V-ATPase-V0a2 Inhibition Enhances Cisplatin-Mediated Cell Death by Elevating Both Intrinsic and Extrinsic Apoptosis

Upon therapeutic stress, the fate of a cancer cell is decided by its apoptotic capacity. Induction of intrinsic apoptosis is the primary mechanism of cisplatin-mediated cell death [20]. To understand the precise mechanism by which V-ATPase inhibition leads to sensitization of cisplatin resistant cells, we performed cell death pathway PCR array using cisplatin treated sh-V0a2-cis-R and sh-scr-cis-R cells. The proapoptotic genes (caspase 3, FASL, caspase 8, TNF, and TNFR1) were significantly upregulated (p< 0.05) in V0a2 knockdown cells upon cisplatin treatment [Supplementary Figure S2]. Flow cytometry analysis revealed that the protein levels of active caspase 3 were increased in sh-V0a2-cisR cells (p=0.009) relative to sh-scr-cisR. The intrinsic apoptotic proteins, active caspase-9 (p=0.02) and Bax (p =0.03) [Figure 2(c)], were also upregulated in sh-V0a2-cis-R. Further, cell membrane-bound FasL and cleaved caspase 8 levels, members of the extrinsic apoptotic pathway, were also elevated [Figure 2(d)] compared to control cells. This indicates that inhibition of V0a2 expression potentiates the cell death activity of cisplatin by stimulating both intrinsic and extrinsic apoptotic pathways.

### 4.4. V-ATPase-V0a2 Inhibition Dampens the Protective Autophagy Levels in Cisplatin Resistant Ovarian Cancer Cells

**Figure 3:**
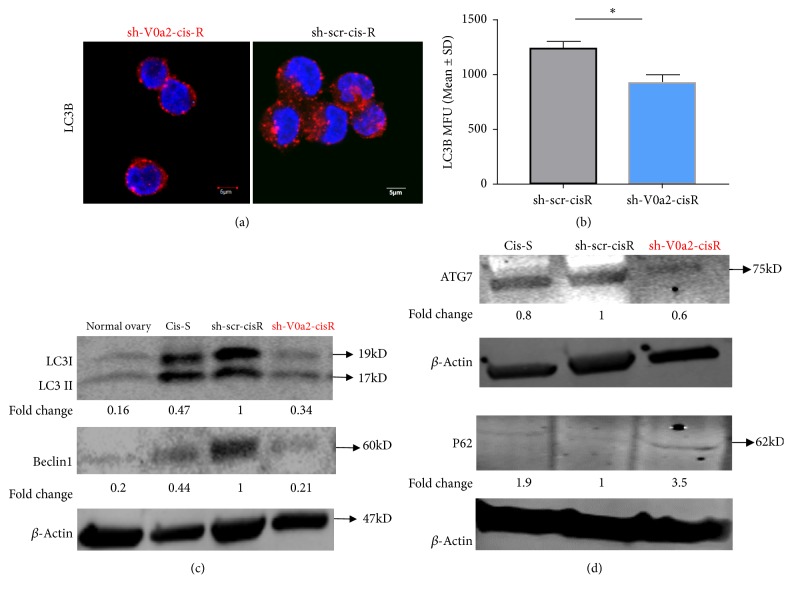
*Protective autophagy is dampened upon V-ATPase-V0a2 inhibition in cisplatin resistant ovarian cancer cells.* (a) Confocal microscopy analysis of the subcellular distributions of LC3B levels (red) in V-ATPase-V0a2 inhibited cisplatin resistant cells (sh-V0a2-cisR) compared to control (sh-scr-cis-R) cells. The sh-V0a2-cisR exhibit low LC3B staining compared to control cells (x600 magnification). (b) Geometric mean fluorescence intensity (MFU) of LC3B levels in sh-V0a2-cisR compared to the levels in control (sh-scr-cisR) cells as quantified by flow cytometry. (c) Western blot analysis of the autophagy associated proteins LC3 (I and II) and Beclin1 in sh-V0a2-cisR shows decreased expression compared to control cells (sh-scr-cis-R), similar to cisplatin sensitive parental OVCA cells (cis-S). (d) Western blot analysis of ATG-7 shows lower expression upon V-ATPase V0a2 inhibition while P62 (autophagy substrate protein) shows higher expression in sh-V0a2-cisR compared to control cells (sh-scr-cis-R). Fold change in band densities were measured relative to the control (sh-scr-cisR) and the samples were normalized to endogenous beta-actin levels. Representative images from three independent experiments are shown here.

An enhanced autophagy process reflects an enhanced survival mechanism in cisplatin resistant cancer ovarian cancer cells [21, 22]. For successful the autophagy process, the proton pumping activity of V-ATPase is necessary for the acidification of the endo-lysosomal vesicles mediated degradative stage [23]. Inhibition of autophagy is known to sensitize the resistant cells to cisplatin treatment [24–26]. We therefore studied the effect of blocking V-ATPase-V0a2 on the modulation of autophagy in chemoresistant cells. In our previous study, we showed that the sh-V0a2-cisR growth rate was slower than the sh-scr-cisR cells; however, V0a2 inhibition in itself did not impose any cytotoxicity to the cisplatin resistant cells. In the context of autophagy, here, we found lower autophagosome numbers in sh-V0a2-cisR compared to sh-scr-cisR cells by confocal microscopy analysis [Figure 3(a)]. Lower autophagosome accumulation was also confirmed by flow cytometry analysis [Figure 3(b)]. Further, a significantly reduced LC3B, Beclin-1 [Figure 3(c)], and ATG7 [Figure 3(d)] levels were observed in sh-V0a2-cis-R compared to control cells as determined by western blot analysis indicating that the initial autophagy steps are inhibited by V-ATPase inhibition. This is in contrast to V-ATPase inhibition using chemical inhibitors which are known to acutely induce autophagy as a protective mechanism. Interestingly, autophagy substrate protein P62 was upregulated in sh-V0a2-cisR, suggesting a concomitant block in the autophagy flux due to interference with endosomal function [Figure 3(d)].

### 4.5. Inhibition of V-ATPase-V0a2 Disrupts Early Endosome Trafficking in Cisplatin Resistant Ovarian Cancer Cells

**Figure 4:**
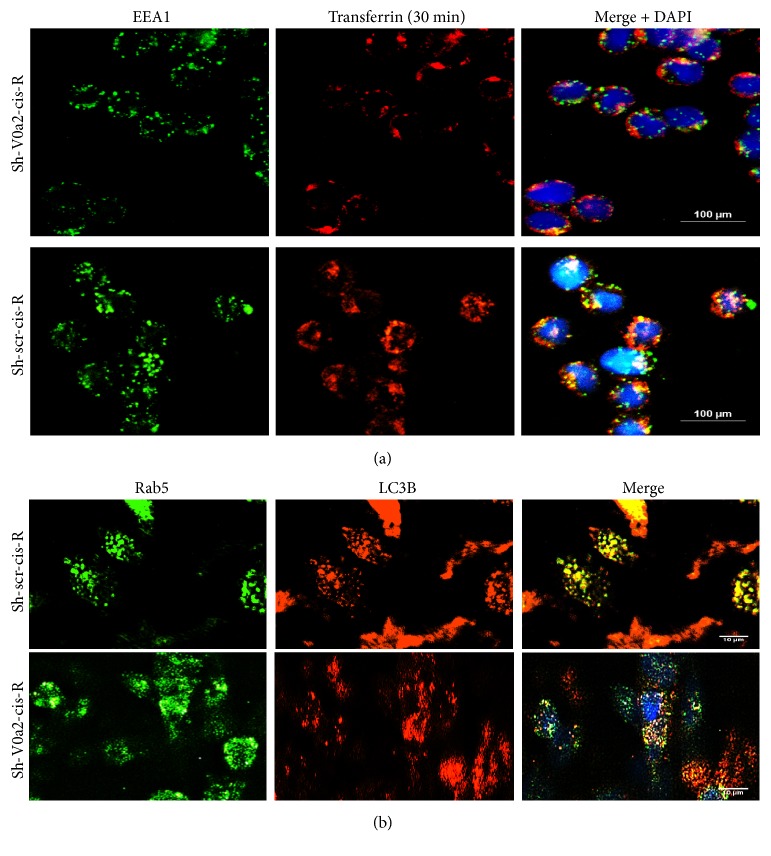
*Inhibition of V-ATPase-V0a2 disrupts early endosome trafficking in cisplatin resistant ovarian cancer cells.* (a) Cisplatin resistant OVCA cells were treated with control shRNA (sh-scr-cis-R) or with shRNA against V-ATPase-V0a2-cisR (sh-V0a2-cis-R). The cells were incubated with Tf-Alexa_594_ at 37°C for 30 min to label the entire early endosomal compartment. The cells were fixed before permeabilization and were stained with anti-EEA1 (green). Merged images of shV0a2-cis-R and sh-scr-cis-R cells. Yellow color indicates colocalization between Tf-Alexa_594_ and EEA1. (b) Immunofluorescence analysis of the subcellular distributions of LC3B (red) and Rab5 (early endosome marker; green) in sh-V0a2-cis-R compared to control (sh-scr-cis-R) cells. The sh-V0a2-cisR exhibited low poor LC3B/Rab5 colocalization compared to control cells (x600 magnification).

The isoform-specific V-ATPase inhibition impairs specific organellar functions in contrast to the chemical V-ATPase inhibitors that target the predominant subunits on cellular V-ATPases. For the formation of autophagolysosomes, autophagic vacuole undergoes maturation through fusion with early/late endosomes and lysosomes [27, 28]. Since V0a2 is primarily localized on the early endosomal membrane to regulate the vesicular pH, we first analyzed the effect of V0a2 knockdown on early endosomal trafficking. To measure the function of the early endosome, we examined transferrin (Tfn) uptake, using Alexa _594_-labeled Tfn. In sh-V0a2-cis-R cells, a 30-min incubation with Tfn showed a surface accumulation and a reduction in the amount of internalized Tfn compared to control sh-scr-cis-R cells [Figure 4(a)]. When the cells (tfn internalized, 30 min) were fixed and stained with EEA1 (early endosome marker), an intense colocalization of Tfn was observed in control sh-scr-cis-R cells. In contrast, sh-V0a2-cis-R cells showed low transferrin signal in early endosomes [Figure 4(a)]. Further, LC3B stained autophagosomes and EEA-1 exhibited diminished colocalization in sh-V0a2-cis-R cells compared to control cells (sh-scr-cis-R) [Figure 4(b)]. The autophagy vacuoles (LC3B) colocalized with the late endosomes/lysosomes in V0a2 depleted cisplatin resistant cells similar to the control cells [supplementary Figure S3]. Further in-depth studies are required to understand the precise role of functional early endosomes in autophagy process.

### 4.6. Cisplatin Treatment in V-ATPase-V0a2 Inhibited Chemoresistant Cells Induces Autophagy

**Figure 5:**
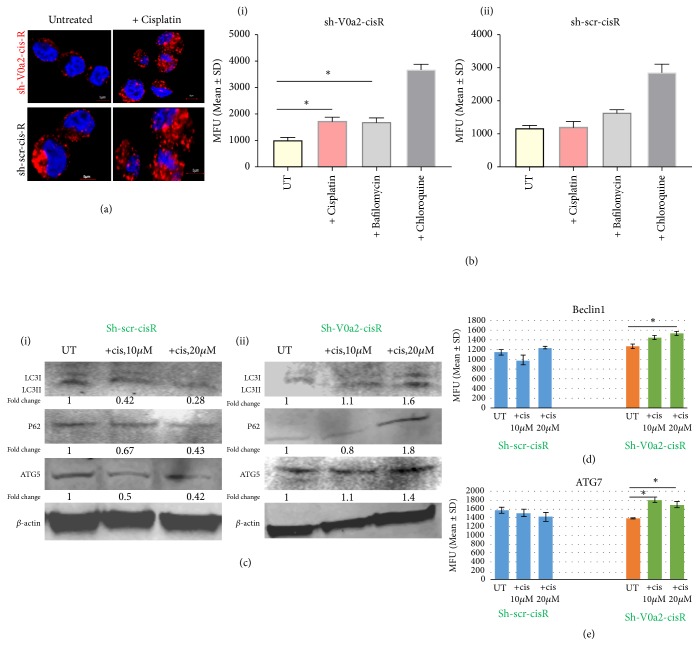
*Cisplatin induces protective autophagy in V-ATPase-V0a2 inhibited resistant ovarian cancer cells with a concomitant block in autophagy flux leading to drug sensitization*. V-ATPase-V0a2 inhibited cisplatin resistant ovarian cancer cells (sh-V0a2-cisR) were treated with cisplatin (20*μ*g/ml, 48h). (a) Confocal microscopy analysis of the subcellular distributions of LC3B levels (red); nucleus is stained with DAPI (blue). Upon cisplatin treatment, there is a higher accumulation of LC3B in sh-V0a2-cisR compared to untreated cells. The fluorescence signals of LC3B were sequentially acquired using an Olympus FluoView confocal microscope. Representative confocal micrographs (original magnification: 80X) are shown. Bars, 5*μ*m. (b) Geometric mean fluorescence intensity units (MFU) of LC3B levels in sh-V0a2-cisR compared to the levels in untreated cells as quantified by flow cytometry. (c) Western blot analysis of the autophagy associated proteins LC3, P62, and ATG5 in (i) control sh-scr-cisR cells and (ii) sh-V0a2-cisR cells upon cisplatin treatment. Geometric mean fluorescence intensity of (d) beclin1 protein levels and (E) ATG7 protein levels in cisplatin treated/untreated sh-V0a2-cisR compared to control cells (sh-scr-cisR) as quantified by flow cytometry.*∗* p<0.05. Experiments were repeated twice in duplicate.

In the parental cisplatin sensitive OVCA cells, autophagy overactivation is a known contributor to cisplatin-mediated cell death [22]. In line with the previous reports, we observed an enhanced autophagy response in cisplatin sensitive OVCA cells (Cis-S) upon cisplatin treatment. There were an increased autophagosome number and decreased P62 levels [Supplementary Figure S4] in cisplatin treated cis-S cells. However, the cisplatin resistant cells are known to depict a protective autophagy response that counteracts apoptotic cell death. Moreover, pharmacological inhibition of autophagy either by inhibiting ATGs, beclin, or lysosomal inhibitors enhances cisplatin-mediated apoptosis [21, 24–26]. Here, sh-V0a2-cisR when treated with cisplatin showed induction of several autophagy initiation related genes such as MAPLC3A, IGFR, and Atg7 [Supplementary Figure S5]. The upregulation in the autophagy initiation proteins was confirmed by flow cytometry. The sh-V0a2-cisR cells exhibited higher autophagosome accumulation upon cisplatin treatment compared to sh-scr-cisR as determined by confocal microscopy analysis [Figure 5(a)]. This enhanced autophagosome accumulation was further confirmed by flow cytometry analysis of LC3B protein in sh-V0a2-cisR and sh-scr-cisR [Figure 5(b)]. x Therefore, upon cisplatin treatment of sh-V0a2-cis-R, in spite of enhanced autophagy initiation, there was a concomitant accumulation of autophagosomes with high accumulation of P62 suggesting an overall block in autophagy flux that facilitates cisplatin-mediated cell death [Figures 5(c)(i) and 5(c)(ii)]. Flow cytometry analysis confirmed upregulated expression of autophagy initiation proteins Beclin-1 and ATG7 [p<0.05; Figures 5(d) and 5(e)].

### 4.7. Enhanced Cisplatin Sensitization upon V-ATPase Inhibition Involves Suppression of ERK/MEK Pathway in Resistant Ovarian Cancer Cells

**Figure 6:**
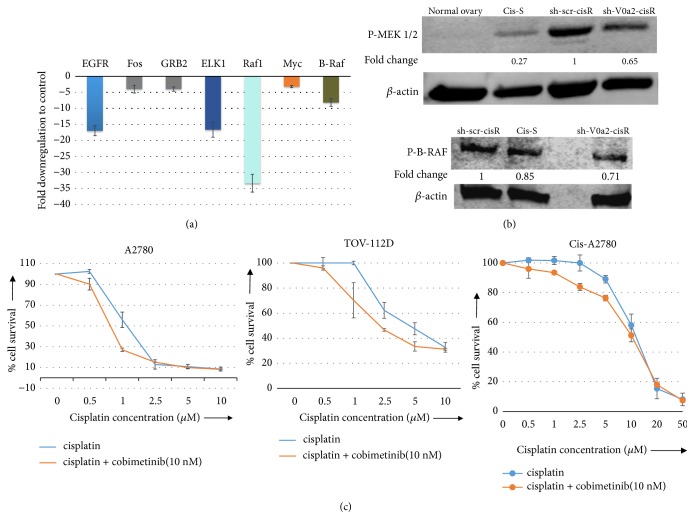
*V-ATPase-V0a2 inhibition sensitizes the cisplatin resistant ovarian cancer cells through downregulation of the Ras pathway.* The shRNA mediated V-ATPase-V0a2 inhibition was carried out in cisplatin resistant ovarian cancer cells (sh-V0a2-cisR). Cisplatin treated (20*μ*g/ml, 48h), sh-V0a2-cisR and control cells (sh-scr-cisR) were analyzed for Ras pathway. (a) Transcriptional profiling of the Ras pathway array showed significant downregulation of the Ras pathway associated genes (EGFR, Fos2, GRB2, Raf1, Elk-1 Myc, and B-Raf). (b) Western blot analysis showed downregulation of phosphorylated B-Raf and MEK 1/2. Fold change in band densities was measured relative to the control (sh-scr-cisR) and the samples were normalized to endogenous beta-actin levels. (c) Combination of cisplatin and MEK inhibitor cobimetinib (10nM) showed enhanced cell death in three ovarian cancer cell lines (A2780, TOV-112D, and cis-A2780).

Upregulation of oncogenic Ras pathway has been observed in chemoresistant cancer cells. The Ras pathway enhances DNA-repair through the Ras/PI3K/Rac1 pathway [29, 30] to protect against cisplatin-induced therapeutic stress. In addition, inhibition of Ras may enhance the cisplatin sensitivity of human glioblastoma [31]. In the present study, the mRNA profiling of the Ras pathway array of the sh-V0a2-cisR revealed significant downregulation of certain Ras pathway genes (EGFR, Fos, BRAF, GRB2, ELK, Raf1, and Myc) [Figure 6(a)]. The western blot analysis confirmed downregulated phosphorylation of MEK1/2 and BRAF, thus confirming the suppression of ERK/MEK pathway in these cells compared to sh-scr-cisR control cells [Figure 6(b)]. Further, the treatment of cisplatin resistant ovarian cancer cells (cis-A2780) with the MEK1/2 inhibitor cobimetinib sensitized the human ovarian cancer cell lines to cisplatin-induced cell death. Cisplatin alone did not elicit significant cell death, whereas enhanced cell death was seen when cells were treated for 48 h with cisplatin in combination with 10nM cobimetinib [Figure 6(c)]. At the same concentration of cobimetinib, an enhanced cell death was observed more prominently in cisplatin sensitive cell lines (A2780 and TOV-S) compared to cisplatin resistant cells (cis-A2780) suggesting a varied activation and role of ERK/MEK pathway in cisplatin resistance. Taken together, these findings indicate that V-ATPase mediated inhibition of autophagy flux contributed to the reversal of cisplatin resistance in resistant ovarian cancer cells. Knockdown of V0a2 or use of MEK inhibitors suppresses ERK activation and blocks the autophagy while increasing cisplatin-induced cell death.

## 5. Discussion

Ovarian cancer is the leading cause of cancer-related deaths in women due to high treatment failure rates [32]. To improve the OVCA patient outcome, it is imperative to understand the chemoresistance associated pathways to identify the mode for sensitizing cancer cells to therapy [20]. The prominent mechanism of chemoresistance includes resistance to apoptosis [33]. Alternative cell survival pathways such as autophagy have become a critical area of research [34, 35]. The process of tumorigenesis exhibits varied dependence on autophagy during progression from primary tumor to metastatic stage or during chemoresistance [36]. The role of autophagy in cancer is therefore highly debatable and reports on the anticancer effects upon autophagy inhibition or its overactivation are equally available [37, 38]. In some cases, overactive autophagy induces cell death necessary for the cytotoxic effect of the therapy. Therefore, autophagy inducers such as the mTOR inhibitors have also been considered as potential cancer treatments [39–41].

The chemoresistant cancer cells, however, exhibit a distinct relation with the autophagy process. Several studies have revealed that cisplatin resistant ovarian cancer cells express high levels of autophagy as a survival mechanism [21, 22]. The fact that anticancer drugs frequently induce cytoprotective autophagy has provided the basis for combination therapy trials using autophagy-blocking agents with standard antitumor drugs [42]. Several clinical trials are investigating the efficacy of autophagy inhibition with conventional chemotherapy in various types of cancers [43]. Given that autophagy is also a basic physiological mechanism in all cells [8, 44], directly targeting the autophagy in cancer leads to unwanted consequences in normal cells. It is therefore vital to identify indirect autophagy modulators specific for cancer cells that can be effectively targeted for anticancer therapy.

Cancer drug resistance is associated with an altered pH gradient between the cytosol and extracellular/intravesicular space, primarily driven by proton pumps V-ATPases [45–47]. This altered pH gradient interferes with drug uptake and metabolism in cancer cells [48]. Regulated assembly of the V-ATPase V0 and V1 domain occurs in response to glucose starvation and involves both PI3K and AMPK pathway, which in turn modulate autophagy [49]. In this context, it will be interesting to examine the long term effect of dysfunctional V-ATPase complex and its effect on AMPK pathway. In the previous studies, we identified V-ATPase-V0a2 as a tumor-associated isoform that is distinctly overexpressed in cisplatin resistant ovarian cancer cells, the inhibition of which sensitized the cells to cisplatin treatment [17, 18]. In continuation with our efforts, in the present study, we demonstrate that the inhibition of V-ATPase-V0a2 sensitizes cisplatin resistant OVCA cells by direct modulation of autophagy process. Cisplatin treatment induces extrinsic and intrinsic apoptotic pathways upon V-ATPase-V0a2 isoform inhibition in resistant OVCA cells with inhibition of protective autophagy. This makes the chemoresistant cells susceptible to any further genotoxic stress. Our data is in line with the previous reports suggesting that inhibition of autophagy sensitizes acquired cis-R cells to cisplatin [21, 22].

**Figure 7:**
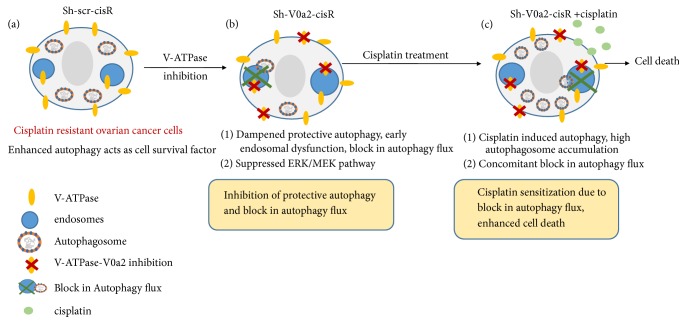
*V-ATPase-V0a2 isoform inhibition mediates cisplatin-associated cell death in resistant ovarian cancer through a block in autophagy flux.* (a) Cisplatin resistant ovarian cancer cells exhibit enhanced autophagy that favors cell survival under stress. (b) Upon V-ATPase inhibition, the autophagy initiation proteins are dampened. There is a block in autophagy flux reflected by high P62 accumulation. Suppressed ERK/MEK pathway is also observed. (c) Cisplatin treatment of V-ATPase inhibited cells triggers autophagy; however, there is a concomitant block in autophagy flux, leading to autophagosome accumulation and enhanced cell death.

Upon cisplatin treatment, sh-V0a2-cisR cells show induction of autophagy pathway as a prosurvival mechanism; however, the accumulation of autophagy substrate P62 confirmed a concomitant block in the overall autophagy flux that drives the cells towards cell death. V0a2 inhibition suppressed the ERK/MEK pathway in cis-R cells. Our findings provide key evidence that the isoform-specific inhibition of V-ATPase-V0a2 inhibits autophagy that contributes to cisplatin sensitization in resistant OVCA cells [Figure 7].

Previous studies have confirmed that V-ATPase proton pump subunits are inducible by cisplatin treatment [50]. This prevents cytosolic acidification of cancer cells that is a trigger of apoptosis [51]. Further, the acquired cisplatin resistant cells show upregulated expression of V-ATPase subunits [50, 52]. V-ATPase driven proton flux causes acidification of intracellular vesicles as well as the acidification of the extracellular microenvironment of cancer cells. This interferes with drug-induced cytotoxicity in addition to promoting cancer invasiveness [53].

In clinical tissues, there are no detailed reports on the correlation between V-ATPase expression and drug responsiveness in ovarian cancer patients. Our study demonstrates for the first time increased expression of V-ATPase-V0a2 in clinical tissues obtained from cisplatin nonresponder patients compared to the treatment-responder patients. The data provided here include samples showing intrinsic unresponsiveness to the treatment. Further studies are therefore required in OVCA tissue samples from relapse/posttreatment patients to improve our understanding of the acquired chemoresistance. Such tools will greatly improve the prediction of sensitivity or resistance to chemotherapy and allow treatment stratification.

Cisplatin resistant cancer cells exhibit defective steps in apoptosis with decreased expression of proapoptotic proteins such as BAD, Bid, and caspases 4 and 6 [3, 20, 54]. Our previous study demonstrated that the apoptotic rate of V0a2 inhibited cisplatin resistance cells was higher than in the control group [18]. Here, we confirm that cisplatin treatment effectively induces both intrinsic and extrinsic apoptosis in resistant OVCA cells upon V0a2 inhibition by the activation of caspase-3, caspase-8, caspase-9, and caspase-7.

However, targeting the apoptotic mechanisms does not optimally inhibit chemoresistance [55, 56]. It is therefore critical to exploit the alternative pathways, such as autophagy to counter chemoresistance. Targeting the autophagy for regulation of cancer chemoresistance is a therapeutic strategy yet to be properly designed. With regard to cisplatin resistance, several reports suggest that protective autophagy inhibition is particularly helpful in chemosensitization [22, 57–59]. Our study confirms that V-ATPase-V0a2 inhibition significantly inhibits autophagy process with downregulation of beclin-1 and LC3 levels compared to control cells. After cisplatin treatment, several early autophagy-related genes were induced in response to drug-induced stress; however, there was a concomitant accumulation of autophagosomes and elevated P62 levels suggesting an overall block in autophagy flux that facilitates cisplatin-mediated cell death.

For successful the autophagy process, a fully functional endo-lysosomal system is highly critical. For autophagic vacuole (AV) maturation, a sequential fusion of AVs with different populations of early and late endosomes and lysosomes [27, 60] is essential which highlights the importance of different vesicles of the endo-lysosomal pathway in autophagy. Chloroquine (CQ) derivative, hydroxychloroquine (HCQ), the only clinically approved autophagy inhibitor, is presently under clinical trials as mono- or combination therapy against various types of cancers [61]. HCQ gets sequestered in the acidic vesicles such as autolysosomes making them alkaline, thereby hindering the degradative steps. However, CQ derivatives are known to produce harmful side effects specifically on heart and kidney. Similarly, bafilomycin is another known chemical that inhibits V-ATPase and disrupts lysosomal acidification and autophagy that contributes to tumor cell death; however, it also interferes with Ca2+ pump SERCA [62]. Proton pump inhibitors (PPI) such as omeprazole and pantoprazole induce the early accumulation of autophagosomes, with concomitant inhibition of autophagic flux [63, 64]. Considering the side effects of the known chemical autophagy and V-ATPase inhibitors, V-ATPase-V0a2 isoform-specific targeting for V-ATPase/autophagy inhibition will provide a safer anticancer alternative. In addition, due to the absence of V0a2 isoform on renal cells (where a4 isoform is predominant), targeted V0a2 based therapy may have fewer renal associated side effects.

Recently, functional early endosomes were found to be essential for successful autophagy. Defects in early endosome function led to an accumulation of autophagosomes and inhibition of autophagy [10]. V0a2 is found in the early endosomes and is also known to regulate protein degradative pathway through interaction with Arf6 and ARNO [65]. V-ATPase inhibition led to dysregulation of Notch signaling due to endosomal acidification [66]. Here, V-ATPase inhibition by V0a2 knockdown indicates defective early endosomal function that may contribute to modulation of autophagy process. Further in-depth studies are required to decipher the exact mechanism of V0a2 mediated regulation of early endosomal function.

The mechanism of chemoresistance is dependent on the balance of the activities of different intracellular signaling systems. There are extensive reports on the involvement of survival signals such as MAP kinase subfamilies in regulating autophagy [67, 68]. Several studies indicate that ERK (extracellular signal-regulated kinase), one of the six known mammalian MAPK pathways, is aberrantly activated in cancer cells and promotes cancer cell proliferation, suppresses apoptosis, and enhances metastasis/drug resistance. ERK (ERK1 and ERK2) is activated upon phosphorylation by MEK (MEK1 and MEK2), which is activated upon phosphorylation by Raf (Raf-1, B-Raf, and A-Raf). ERK pathway activation promotes autophagy, leading to cisplatin resistance. Inhibition of ERK activation enhances cisplatin-induced growth inhibition [69]. In cisplatin resistant squamous cancer cells, both ERK (extracellular signal-regulated kinase) activation and autophagy induction are observed [70]. A recent report suggests that V-ATPase regulates not only endosomal receptor recycling, but also the lipid composition of the plasma membrane which is crucial for the activation of Ras [71]. In the present study, in line with the previous observations, we observed suppression of activated MEK1/2 and B-RAF in V-ATPase-V0a2 inhibited cisplatin resistant OVCA cells compared to control. The combination treatment of small molecular MEK1/2 inhibitor, cobimetinib along with cisplatin, could enhance cisplatin-mediated cell death in OVCA cells. The combination of MEK inhibitor with cisplatin, however, is more effective in sensitive cells compared to resistant cells, indicating that there are other significant players in chemoresistance.

Taken together, these findings indicate that inhibition of autophagy contributes to the reversal of cisplatin resistance in ovarian cancer cells. From the results obtained in this study, we propose that targeting V-ATPase-V0a2 is an effective strategy in sensitizing the chemoresistant ovarian cancer cells to cisplatin treatment.

## Data Availability

The data used to support the findings of this study are available from the corresponding author upon request.

## Supplementary Materials

Supplementary Materials The supplementary material provided with the manuscript contains Supplementary Figures S1-S5 along with the figure legends.
