# Supplementary material for: Targeting V-ATPase Isoform Restores Cisplatin Activity in Resistant Ovarian Cancer: Inhibition of Autophagy, Endosome Function, and ERK/MEK Pathway
Source: J Oncol. 2019 Apr 1;2019:2343876. doi: 10.1155/2019/2343876 (PMC6463777; doi:10.1155/2019/2343876)
Supplement: Supplementary Materials — The supplementary material provided with the manuscript contains Supplementary Figures S1-S5 along with the figure legends. [file 2343876.f1.docx]

**Supplementary figures**

**Supplementary figure S1:** V-ATPase-V0a2 is expressed on cancer cells in cisplatin responder ovarian cancer patient tissues.

**
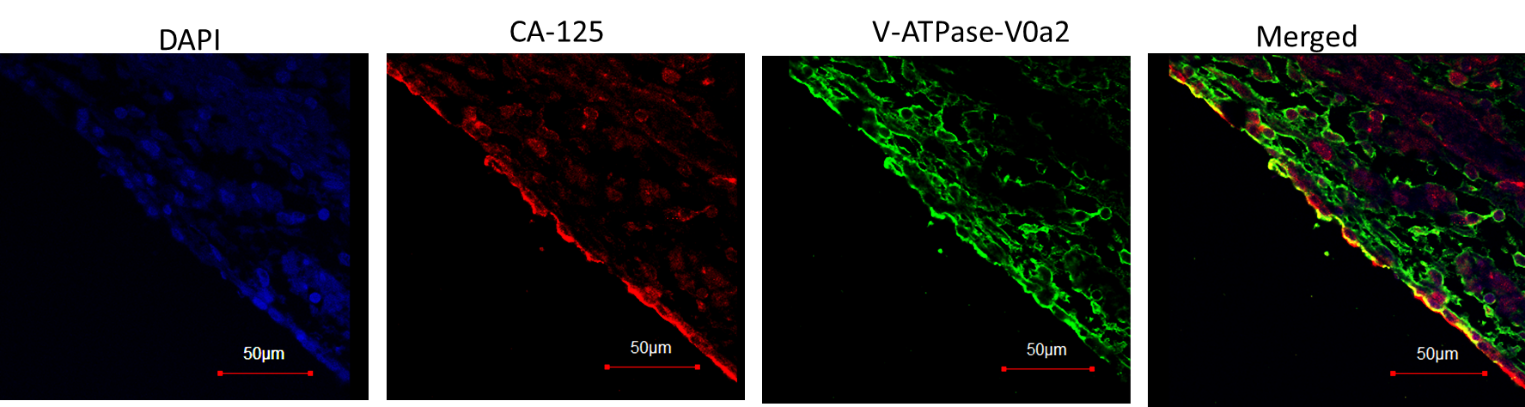
**

**Figure S1 legend:** Confocal microscopy analysis of V0a2 (green) in drug responder OVCA tissues showing its co-expression with ovarian cancer cell marker CA125 (red). Nuclear DAPI staining in blue. Merged areas shown in yellow. Original magnification: × 600.

**Supplementary figure S2:** Cisplatin treatment induces apoptotic cell death pathway related genes in V-ATPase-V0a2 knockdown resistant ovarian cancer cells.


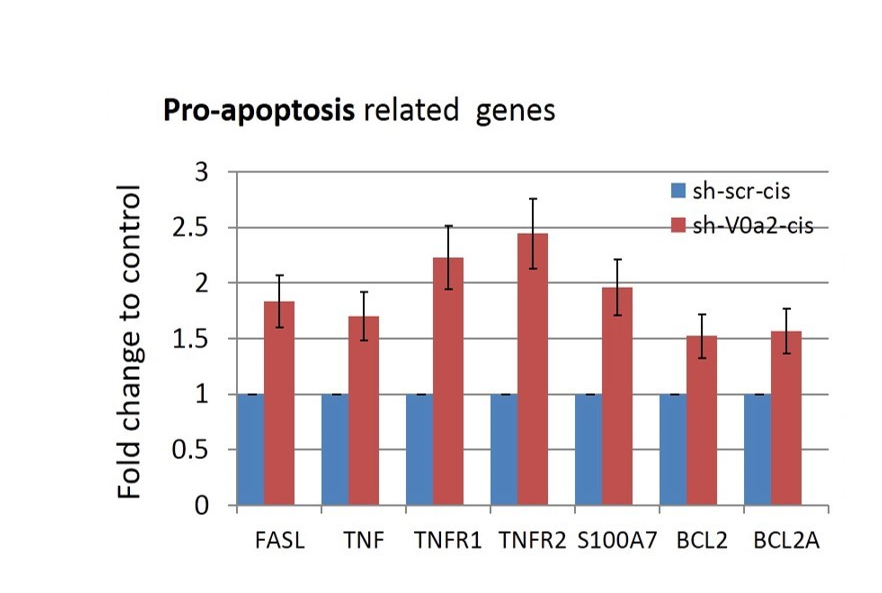


**Figure S2 legend**: Figure legend: Q-RT-PCR array was performed for identification of the pathways involved in cisplatin mediated cytotoxicity in V-ATPase-V0a2 knock down cells. The cell death pathway related genes belonging to pro-apoptotic mechanism were found to be elevated. Two endogenous controls (GAPDH and HPRT) were used for the analysis. Mean ±SD of two independent observations are depicted here.

**Supplementary figure S3:** VATPase-V0a2 inhibition does not affect the fusion of autophagosome with late-endosome/lysosome

**
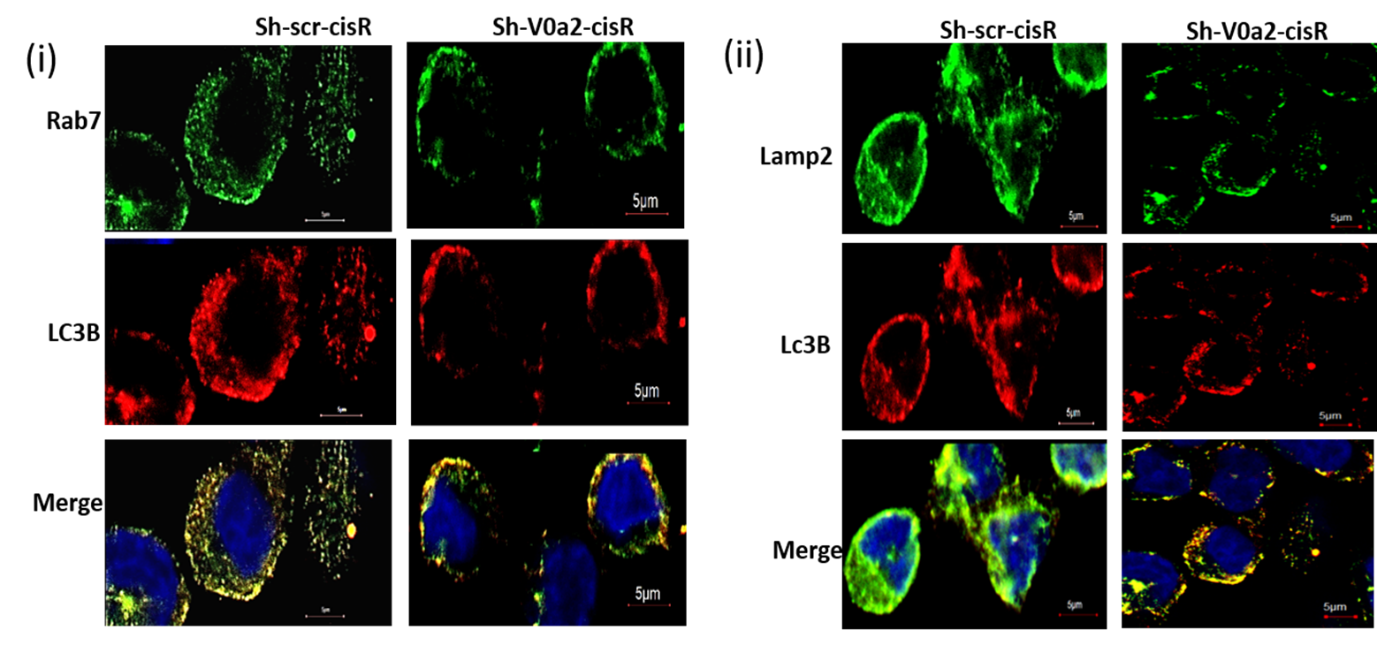
**

**Figure S3 legend**: Confocal microscopy analysis of cisplatin resistant ovarian cancer cells (sh-scr-cisR) and VATPase-V0a2 inhibited counterparts (sh-V0a2-cisR). Cells were stained for LC3B, Rab7 and Lamp-2. (i) sh-V0a2-cisR and sh-scr-cisR showed LC3B staining (red) that co-localized with Rab7 293 (green). Merged areas shown in yellow, lower panel. (ii) Similarly, sh-V0a2-cisR and sh-scr-cisR showed LC3B staining (red) that co-localized with Lamp2 (green). Merged areas shown in yellow, lower panel. Magnification X600.

**Supplementary figure S4:** Parental cisplatin sensitive ovarian cancer cells (A2780) exhibit autophagy over-activation as mechanism of cisplatin mediated cell death

**
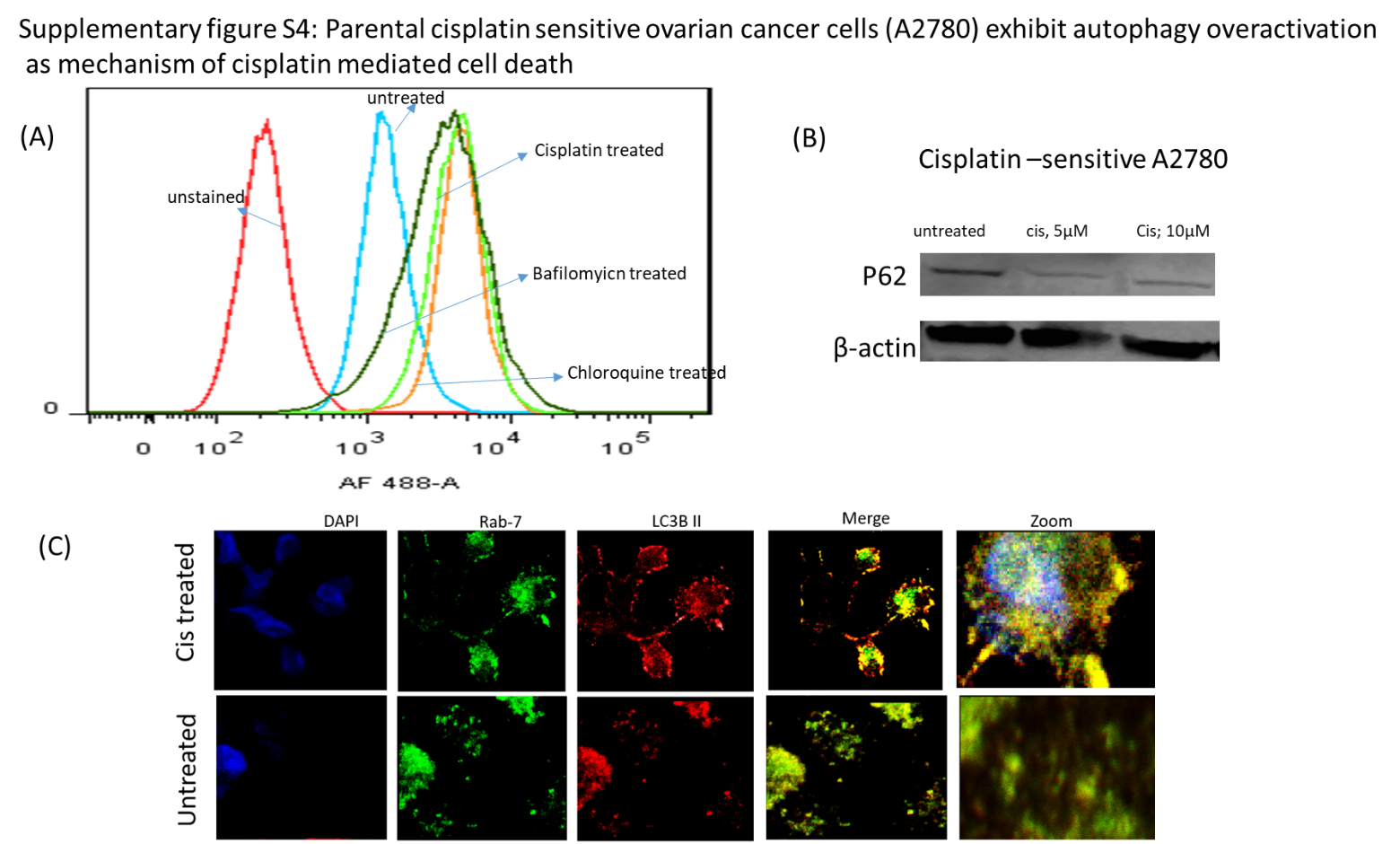
**

Figure S4 legend: (A) Flow cytometry analysis of LC3B containing autophagosome (green) in cisplatin sensitive parental OVCA cells (A2780). There is enhanced autophagosome numbers in cisplatin treated cells (bright geen line) compared to untreated control cells (blue line). (B) Western blot analysis of the autophagy substrate protein, P62, showing decreased expression in cisplatin treated A2780 cells, indicating an over-activated autophagy levels as a mechanism of cell death. (C) Autophagosomes colocalize with the acidic endosomes in cis-S cells.

**Supplementary figure S5:** Cisplatin treatment induces autophagy pathway related genes in V-ATPase-V0a2 knockdown resistant ovarian cancer cells.


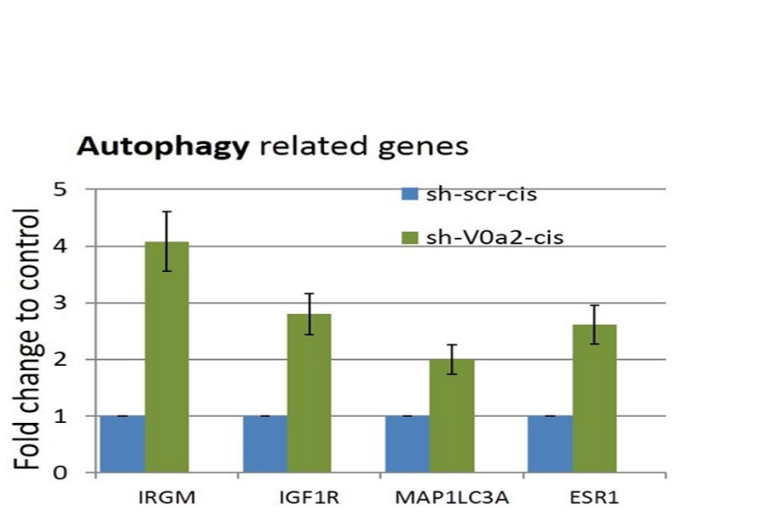


Figure S5 legend: Q-RT-PCR array was performed for identification of the pathways involved in cisplatin mediated cytotoxicity in V-ATPase-V0a2 knock down cells. The genes belonging to autophagy mechanism were found to be elevated. Two endogenous controls (GAPDH and HPRT) were used for the analysis. Mean ±SD of two independent observations are depicted here.
